# O04 Parental preferences for primary care pathways in respiratory infections: a discrete choice experiment

**DOI:** 10.1093/jacamr/dlag102.004

**Published:** 2026-06-26

**Authors:** Anthony Maher, Eimear C Morrissey, Andrew W Murphy, Sharon Walsh, Gerard J Molloy

**Affiliations:** School of Psychology, University of Galway, Galway, County Galway, Ireland; Centre for Health Research Methodology, School of Nursing and Midwifery, University of Galway, Galway, County Galway, Ireland; Department of General Practice, College of Medicine, Nursing and Health Sciences, University of Galway, Galway, County Galway, Ireland; HRB Primary Care Clinical Trials Network, University of Galway, Galway, County Galway, Ireland; Health Economics and Policy Analysis Centre, J.E. Cairnes School of Business and Economics, University of Galway, Galway, Ireland; School of Psychology, University of Galway, Galway, County Galway, Ireland

## Abstract

**Background:**

Antimicrobial resistance is a global One Health challenge. It is particularly relevant to respiratory tract infections (RTIs) in primary care, where antibiotics are often prescribed despite many infections being self-limiting. Stewardship strategies targeting high-use groups such as children have focused on clinician behaviour, with comparatively less attention to parents. However, parental decision-making may also influence healthcare use and treatment choices, and the trade-offs underpinning these decisions are not well understood.

**Objectives:**

To quantify parental preferences for healthcare pathways and antibiotic prescribing in childhood RTIs.

**Methods:**

A discrete choice experiment (DCE) was conducted among parents in Ireland (*n*=469). Participants completed 12 choice tasks, selecting between two hypothetical healthcare options and an opt-out. Attributes included waiting time, provider type, antibiotic prescribing plan, advice, and cost. Data were analysed using alternative-specific conditional logit models. Willingness-to-pay estimates were derived, and pre-specified interaction terms were used to explore observed preference heterogeneity.

**Results:**

Parents demonstrated strong preferences for shorter waiting times, GP-led care, and lower out-of-pocket costs. Utility decreased with increasing waiting time, with the greatest disutility observed for delays of 72 h (β=−1.18, *P*<0.001). Relative to no antibiotic prescribing, both immediate (β=0.78, *P*<0.001) and delayed (β=0.47, *P*<0.001) prescribing were positively associated with choice. Provision of self-care advice with when-to-worry guidance also increased utility (β=0.54, *P*<0.001). Non-GP providers were associated with lower utility, particularly pharmacist-led care (β=−0.78, *P*<0.001). Cost was negatively associated with utility (β=−0.016, *P*<0.001). Interaction analysis indicated that subsidized healthcare status modified cost sensitivity, location influence provider preferences, and prior antibiotic experience affected antibiotic-related preferences.

**Conclusions:**

Parental preferences for healthcare pathways in childhood RTIs may be shaped by structural and behavioural factors, including access, cost, and the need for reassurance. While antibiotics were positively valued, advice provision and delayed prescribing also contributed to utility, suggesting that antibiotic demand may reflect responses to uncertainty and perceived risk, rather than a fixed preference for medication. Interventions that reduce uncertainty and enhance reassurance may support antimicrobial stewardship without undermining parental confidence in care.

